# Progress in Metabonomics of Type 2 Diabetes Mellitus

**DOI:** 10.3390/molecules23071834

**Published:** 2018-07-23

**Authors:** Quantao Ma, Yaqi Li, Min Wang, Ziyan Tang, Ting Wang, Chenyue Liu, Chunguo Wang, Baosheng Zhao

**Affiliations:** 1 School of Chinese Material Medica, Beijing University of Chinese Medicine, Beijing 100029, China; maquantao@bucm.edu.cn (Q.M.); liyaqi@bucm.edu.cn (Y.L.); weme9916@163.com (M.W.); tangziyan1997@163.com (Z.T.); liuchenyue633@163.com (C.L.); 2 National Center for Safety Evaluation of Drugs, National institutes for Food and Drug Control, Beijing 100176, China; 15011437476@163.com; 3 Beijing Research Institute of Chinese Medicine, Beijing University of Chinese Medicine, Beijing 100029, China; wangcg1119@126.com

**Keywords:** type 2 diabetes mellitus, metabonomics, biomarkers, metabolic pathways

## Abstract

With the improvement of living standards and a change in lifestyle, the incidence of type 2 diabetes mellitus (T2DM) is increasing. Its etiology is too complex to be completely understand yet. Metabonomics techniques are used to study the changes of metabolites and metabolic pathways before and after the onset of diabetes and make it more possible to further understand the pathogenesis of T2DM and improve its prediction, early diagnosis, and treatment. In this review, we summarized the metabonomics study of T2DM in recent years and provided a theoretical basis for the study of pathogenesis and the effective prevention and treatment of T2DM.

## 1. Introduction

With the development of the economy and the improvement of people’s living standards, the incidence of diabetes is increasing year by year. In 2017, the eighth edition of the International Diabetes Federation (IDF) Diabetes Atlas showed that about 425 million of diabetic patients worldwide. It is estimated that the number of diabetic patients would increase to 700 million by 2045 [1]. The incidence of diabetes in China is about 10% [2] and the number of patients has reached 114 million, which is one-third of the total number of diabetic patients in the world and T2DM accounts for more than 90% of the total number of diabetes patients.

T2DM is related to insulin resistance (IR) and islet β cell dysfunction, but the specific mechanisms of its etiology and development are still not fully understood. The clinical indicators such as fasting blood glucose two hours postprandial blood glucose, glycated hemoglobin, and IR are widely used in the diagnosis and classification of T2DM [3], but, when these abnormalities are detected, T2DM may have developed for many years and it might be accompanied with complications. Therefore, finding the biomarkers of diabetes is of great importance, which can help us to further understand the pathogenesis of T2DM and provide a theoretical basis for its risk assessment, early diagnosis, and prevention strategies.

Metabonomics is an omics technology developed after genomics and proteomics, which can simultaneously analyze multiple metabolites in body fluids. The development of metabonomics technology provides possibilities for the prediction and early diagnosis of T2DM. Compared to genomics and proteomics, metabonomics is commonly used in NMR and MS analysis, which has the characteristics of rapid accuracy, high resolution, high sensitivity, and a lower sample size. Therefore, metabonomics is very helpful in discovering the pathophysiological changes of cells, tissues, and body fluids. It is an effective means of discovering disease-related biomarkers, which are more reliable and guaranteed than that of genomics and proteomics [4]. In recent years, some scholars have used metabonomics techniques to conduct a comprehensive and systematic study of metabolite changes in T2DM patients as well as search for the biomarkers and the possible metabolic pathways to provide a theoretical basis for the pathogenesis of T2DM. Metabonomics has also been used to explore the effects of metformin on metabolites in T2DM patients and to explore the metformin’s metabolic mechanism of a hypoglycemic effect [5]. To fully understand the pathological mechanisms of diabetes and provide ideas and references for the future study of the diabetic pathology mechanism, we reviewed the modern achievements of metabonomics technologies in the study of the pathological mechanism of diabetes and look forward to its future development.

## 2. The Pathogenesis of T2DM

### 2.1. IR

IR is an important cause of T2DM, which mainly refers to the decrease in the body’s biological effects on certain concentrations of insulin. The abilities of glucose uptake and metabolism of the body were impaired including the decrease of insulin sensitivity and the decline of the responsiveness [6]. At present, IR is believed to be induced by receptor loss, gene mutations, mitochondrial dysfunction of skeletal muscle [7], and the actions of cytokines such as free fatty acids, tumor necrosis factor, leptin, resistin, and adiponectin.

### 2.2. Impaired Function of Islet Cells

Impaired islet cell function is associated with islet α and β cells. The number of islet β cells is significantly reduced in T2DM patients and the ratio of α/β cells is significantly increased. In addition, the sensitivity of α cells to glucose is decreased, which makes the glucagon level and liver sugar output increase and eventually leads to the incidence of T2DM [8]. This is the classic theory of double hormone abnormalities [9].

### 2.3. Oxidative Stress

IR disrupts glucose and lipid metabolism in the body, which results in the increase of free fatty acids. Excessive free fatty acids stimulate the body to produce large amounts of reactive oxygen species (ROS) and reactive nitrogen species (RNS), which result in oxidative stress [10]. Oxidative stress activates the nuclear factor-κB (NF-κB) signaling pathway by disrupting the mitochondrial structure and inducing apoptosis, which causes cellular inflammatory responses and inhibits insulin synthesis and secretion. Oxidative stress also interferes with physiological processes associated with insulin signaling including the phosphorylation of insulin receptor (InsR) and insulin receptor substrate (IRS), activation of phosphatidylinositol 3-kinase (PI3K), and glucose transport of protein 4 (GLUT4) to produce IR. Oxidative stress also damages the cytoskeleton and eventually produces T2DM [11].

### 2.4. Gene Factors

T2DM has a significant genetic predisposition. Research shows that T2DM is a polygenic hereditary disease. There are many candidate genes identified for T2DM such as *TCF7L2* gene, *KCNQ1* gene, *PPAR-γ* gene, *MODY* gene, *KCNJ11* gene, *Calpain-10* gene, *ENPP1* gene, and *ADIPOR2* gene [12]. The *TCF7L2* gene acts as a transcription factor for the glucan gene and can affect glucagon-like pep-tide1 (GLP-1) levels. GLP-1 has stimulating effects both on insulin secretion and on β-cell growth [13]. The *KCNQ1* gene is expressed in the islets of Langerhans, which selectively blocks K^+^ channels and stimulates insulin secretion. Therefore, *KCNQ1* gene mutation will reduce insulin secretion and affect normal blood sugar regulation [14]. The *PPARγ* is specific for adipose tissue where it plays a key role in regulating adipogenic differentiation [15]. The *Calpain-10* gene has also been shown to inhibit protease involved in a mitochondrial function, which might relate to mitochondrial dysfunction. This is often observed in T2DM [16].

### 2.5. Obesity

Most patients with T2DM are obese especially centrally obesity, which is mainly characterized by abnormal glucose and lipid metabolism. There is a significant negative correlation between the abdominal fat area and insulin-mediated glucose utilization. Central obesity patients have increased abdominal fat, metabolic disorders in visceral adipose tissue (VAT) and impaired inhibition of hepatic glycogen production by insulin leads to IR [17]. An increase in free fatty acids in the liver and muscle leads to the accumulation of lipid metabolites. These lipid metabolites can cause dyslipidemia, impaired β cell secretion of insulin, and excessive fatty acids prevent glucose from clearing, which leads to T2DM [18].

**Figure 1:**
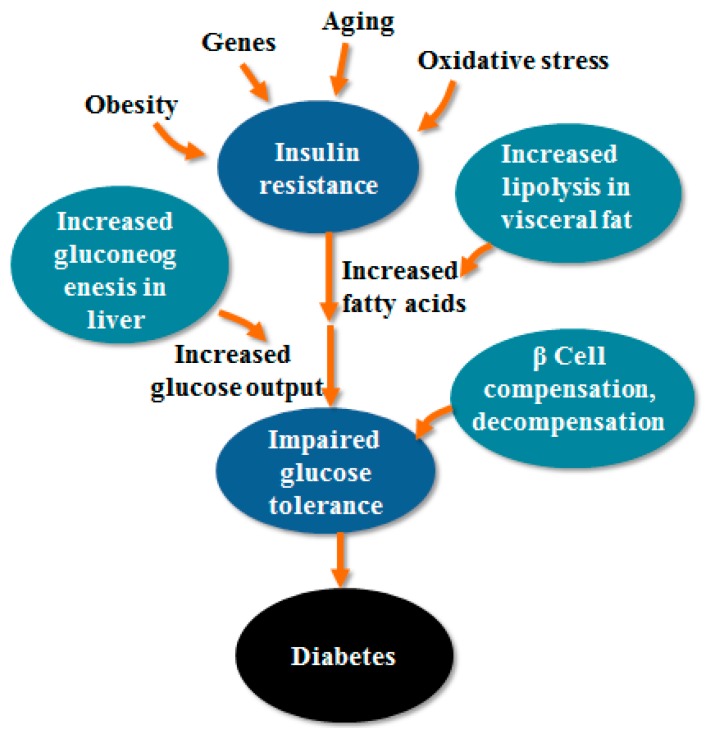
Pathogenesis of T2DM.

The above pathogenesis is shown in Figure 1 [19]. The main feature of T2DM is the decrease of insulin sensitivity. The main causes of T2DM are obesity, oxidative stress, gene and aging, insulin resistance occurs first. The increase of visceral fat leads to the increase of fatty acids, which leads to the increase of gluconeogenesis and glucose levels. The increase of glucose level leads to decompensation of β cells, compensation and decompensation of β cells ultimately leads to impaired glucose tolerance, leading to the development of T2DM.

### 2.6. Other Pathogenesis

#### 2.6.1. Inflammation

In recent years, a growing number of studies have shown that T2DM is a natural immune and inflammatory disease and it is a “low-grade chronic inflammation state.” It is believed that inflammation and immunity are associated with the incidence of T2DM [20]. Inflammatory factors include immune-inflammatory cells (such as white blood cells associated with acute inflammation), acute-phase reaction proteins (such as C-reactive protein, CRP), inflammatory factors (such as tumor necrosis factor, TNF, IL, adiponectin, and resistin), Macrophage Migration Inhibitory Factor (MIF), coagulation factors, lipid components, and others such as sialic acid and amyloid [21,22,23]. Inflammation factors activate intracellular serine/threonine kinases and catalyze the inhibitory phosphorylation of key proteins of the insulin signaling pathway, which leads to IR [24]. Cytokines and chemokines produced by macrophages cause local and systemic inflammation, which, in turn, leads to the dysfunction of islet β cell and IR in liver, fat, and skeletal muscle tissue [25].

#### 2.6.2. Hepatitis B Virus

The Hepatitis B virus (HBV) is closely related to the pathogenesis of T2DM. Custro et al. [26] found that the incidence of T2DM in chronic hepatitis B (CHB) patients is 25%, which is four times that of the general population. Soverini et al. [27] found that HBV infection can increase the susceptibility of T2DM. The above studies have suggested that HBV infection is an important risk factor for T2DM.

## 3. Metabonomics Research Methods and Techniques

Metabonomics is an emerging discipline that has developed rapidly following genomics, transcriptomics, and proteomics. It is mainly responsible for the qualitative and quantitative analysis of small molecules with a relative molecular mass of less than 1000 produced by biological systems containing cells, tissues, fluids, and organisms that are suffering external stimuli or disturbances such as genetic variation or environmental change [28]. Its research process generally includes sample collection, sample preparation, sample analysis, data collection, data preprocessing, multivariate statistical analysis, identification of possible markers, search for metabolic pathways, and biological elucidation.

**Figure 2:**
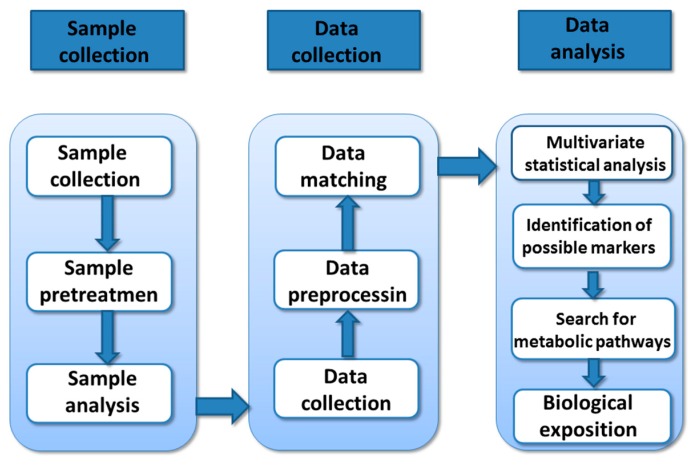
Process flow chart of metabonomics research.

Biological samples (urine, blood, tissues, saliva, cells, etc.) were collected for the inactivation of biological reactions and pretreatment. Then the nuclear magnetic resonance spectroscopy (NMR), mass spectrometry (MS), or chromatographic techniques were used to detect the type, number, status, and changes of metabolites and to obtain metabolic profiles. Then multivariate statistical analysis methods were used to reduce the dimensionality of the acquired data and extract information., The metabolic pathways and changes involved in the metabolites in order to elucidate the response mechanism of organisms correspond to metabolic stimuli and find biomarkers. The specific process is shown in Figure 2.

At present, there are many techniques applied to metabonomics, NMR, and MS are the most commonly used ones.

### 3.1. NMR

NMR is one of the earliest and most widely used techniques in metabonomics research. ^1^H-NMR is the most commonly used. The advantages of the NMR method are outlined below.

(1) The NMR method hardly requires sample pretreatment, enables non-invasive and unbiased detection of the sample, and has good objectivity and reproducibility [29].
(2) NMR is almost non-destructive to the sample (the stability of some samples is limited), which can be used for in vitro [29].
(3) Peaks in the NMR spectrum can represent a certain metabolite, which means an NMR spectrum can provide qualitative and quantitative information on a large number of metabolites in the organism.
(4) ^1^H-NMR responds to compounds containing H, which can complete the detection of most metabolites and meet the goal of detecting as many metabolites in metabonomics as possible.
(5) High-flux NMR techniques with the use of automated liquid handling procedures takes only a few minutes to detect large amounts of metabolite information [30].
(6) Although NMR spectrometers and its recurring expenditures are expensive, NMR is very informative, the cost of a single sample is low, and ultimately the cost of analysis is generally reduced.
(7) NMR can provide rich molecular information including metabolite composition, concentration, molecular dynamics, interactions, pH, and structure.

Frankly, NMR has its disadvantages such as low sensitivity (that is no information may be detected for very small samples) and resolution, which can cause signal overlap. This results in low-abundance analytes being masked by high-abundance analytes. The problems mentioned above are not easily solved due to the limitations of the method itself.

### 3.2. MS

For one thing, the advantage of MS is its higher sensitivity and specificity than NMR. It is an effective tool for studying the molecular structure and detecting and quantifying metabolites. Contrarily, for another, the disadvantages of MS are also clear, which is shown below.

(1) A large amount of sample preparations is required, which are destructive to the sample and, therefore, cannot be studied in vivo or in situ.
(2) There is a need for knowledge of the sample and high recurring costs and the equipment is also quite expensive.

In general, with the development of mass spectrometry and its associated technologies, chromatography-mass spectrometry is increasingly being applied in metabonomics. Combined techniques such as gas chromatography-mass spectrometry (GC-MS) and liquid chromatography-mass spectrometry (LC-MS) methods are commonly used. GC-MS with high resolution and detection sensitivity can be used for qualitative metabolites. The disadvantage is that it requires pretreatment and is cumbersome. LC-MS can be used to analyze compounds with higher relative molecular mass and poor thermal stability. It has high sensitivity and a wide detection range. Therefore, it is more suitable for the detection and identification of potential markers from complex metabolites in biological samples [31,32]. The advantages and disadvantages of NMR and MS are shown in Table 1.

## 4. Advances in the Pathogenesis of T2DM Based on Metabonomics

With the rise of metabonomics, metabonomics research techniques are widely used in the study of various diseases. According to the Human Metabolome Database, ≥2700 endogenous compounds have been detected and quantified in the blood and ≥1400 compounds have been detected and quantified in urine. However, only 87 metabolites related to T2DM were detected and quantified in blood and urine [33]. In recent years, more and more scholars studied the metabolites of T2DM and determined possible biomarkers and their metabolic pathways based on the differences in metabolites providing an important theoretical basis for the effective prevention and treatment of T2DM.

### 4.1. Biomarker

There are two major kinds of factors that can regulate insulin secretion in the human body. One factor refers to nutrients such as glucose, amino acids, and fatty acids. The other factor includes neurotransmitters and hormones. Islet cells maintain a certain homeostasis in different states by coordinating and integrating these two kinds of regulatory factors. T2DM can cause a large number of metabolic abnormalities of substances in the body such as lipids, carbohydrates, amino acids, and more. The abnormalities of these metabolites can be detected by metabolomic techniques to identify early biomarkers that can predict the occurrence and development of diabetes. These biomarkers are important for the study of prevention and treatment of diabetes. A large number of studies have shown that metabolites that changed significantly after the onset of T2DM are mainly amino acids, lipids, and carbohydrates.

#### 4.1.1. Biomarkers Related to Amino Acid Metabolism

Studies have confirmed that amino acids are potential biomarkers of T2DM and branched chain amino acids (BCAA), which include valine, leucine, and isoleucine. These are essential amino acids for human beings. More than 70 years ago, BCAA has been found to be closely related to IR and diabetes [34,35,36]. Newgard et al. [37] found that BCAA contributes to the development of obesity-related IR. A metabolomic study of 74 obese subjects and 67 lean subjects showed that isoleucine, leucine, and valine are associated with IR. They had a significant increase in obese subjects compared with healthy subjects. Further studies indicate that BCAA is significantly associated with IR in both obese and normal weight subjects [38,39]. Recently, Guaschferré et al. [40] analyzed metabolites in up to 8000 individuals (including 1940 patients with T2DM) and found that the increase of isoleucine, leucine, and proline led to an increased relative risk of T2DM. Tyrosine and phenylalanine also had similar effects while glycine and glutamine were negatively correlated with the onset of T2DM. 

Wang et al. [41] followed 2422 non-diabetic individuals for 12 years and found that BCAA and aromatic amino acids (tyrosine, phenylalanine) in non-diabetic patients may be important indicators for the occurrence of diabetes. Their study showed a correlation between BCAA and diabetes. Menni et al. [42] used metabonomics methods to investigate a large sample of people and found that 3-methyl-2-oxolate, which acts as the degradation product of BCAA, was a predictor of impaired glucose tolerance. However, some studies pointed out the level of BCAAs in serum and urine of diabetic patients is decreased [43,44] likely because T2DM patients studied in the above metabonomics studies are not newly diagnosed or untreated. Those selected T2DM patients had elevated serum proline in the study of Bao et al. [45]. Therefore, the results observed in the above studies [40,41] may not reflect the physiological changes of BCAA in T2DM patients [46]. In a large prospective study, Merino et al. [47] followed up with 1150 people with normal fasting blood glucose for 20 years. In addition, 95 of them suffered from T2DM. They found that the decrease of glycine level and the increase of taurine level were closely related to the occurrence of T2DM. The nitrogen metabolism pathway and its components may be potential markers of pathological changes in early stages of T2DM.

Floegel et al. [48] observed a positive correlation between glycine and insulin sensitivity in the study sample. Wang-Sattler et al. [49] used metabonomics technologies to find that the glycine level was a negative predictor of impaired glucose tolerance and diabetes. Their study also found that the glycine-related enzyme 5-aminolevulinic acid synthase 1 (ALAS-H) was up-regulated in patients with T2DM. This means that serum glycine is negatively correlated with the risk of developing T2DM and coupled with the link between insulin and ALAS-H expression [50]. It is speculated that the reduction of serum glycine in T2DM patients may be related to IR. A large prospective study by Wittenbecher showed that red meat intake is strongly associated with the onset of T2DM when serum glycine levels are reduced. The possible mechanism is that the intake of red meat activates the glycine-dependent pathway. Glycine is involved in the synthesis of glutathione, which, in turn, responds to oxidative stress and IR [51]. The glycine intermediate metabolite beta-hydroxypyruvate is also considered to be a predictor of diabetes and β-hydroxypyruvate alters the excitability of intermuscular neurons and decreases the amount of insulin in mice. It was found that the proportion of β-hydroxypyruvate-serine in subjects with impaired glucose tolerance was greatly reduced when compared with subjects with normal ones [52].

Wang et al. [53] performed metabonomics studies on 188 diabetics and 188 non-diabetics from 2422 subjects and found a strong correlation between 2-aminoadipate (2-AAA) and T2DM. A high concentration of neutral amino acids induces protein synthesis and inhibits proteolysis and decreases glucose uptake and glycogen synthesis, which inhibits glucose uptake. These processes carry out protein synthesis and insulin-mediated glucose uptake via the m-TOR signaling pathway [38]. Studies have confirmed the increase of 2-hydroxybutyrate (2-HB) in T2DM patients [54,55,56]. 2-HB synthesis glutathione released as a by-product when cystathionine is cleaved to cysteine and increased homocysteine transferase activity in oxidative stress, which may lead to higher levels of 2-HB and cystine in T2DM patients [57,58].

LU et al. [59] found that 2-HB concentration in serum was positively correlated with Hb A1c (r = 0.420, *p* < 0.001), which was similar to that of Fiehn et al. [54] (r = 0.455, *p* = 0.001). 2-HB may serve as a biomarker of blood glucose control and oxidative stress [60]. Ferrannini et al. [61] found that α-hydroxybutyric acid is a positive predictor of poor glycemic control and diabetes development in a prospective study. Gall et al. [55] found that α-hydroxybutyric acid is the most significant metabolite associated with IR. α-HB, which is a byproduct of α-ketobutyric acid, can identify IR and insulin-sensitive subjects. It may also be a good diagnostic tool when used in conjunction with other biomarkers [54].

Cheng et al. [62] selected over 1000 subjects without diabetes from the cardiac study cohort as subjects and found that there was a significant negative correlation between fasting glutamine concentration and IR. Multivariate logistic regression analysis of the information at the 12-year follow-up of the cohort showed a strong correlation between glutamate and IR. The increase of glutamine/glutamate ratio can predict the risk of diabetes reduction. These potential biomarkers are important for the early diagnosis and therapeutic evaluation of T2DM.

#### 4.1.2. Biomarkers Related to Lipid Metabolism

Diabetes is often accompanied by dyslipidemia [63], but the exact mechanism is not yet clear. Experiments by Krauss et al. [64,65] showed that free fatty acids may be the main causes of IR. LU J et al. [59] used metabonomics to detect significant increases in blood fatty acid levels in patients with T2DM. Mozaffarian et al. [66] used metabolic profile analysis methods to detect fatty acids and phospholipids in more than 3600 non-diabetic subjects. The study found that increased palmitic acid concentration was associated with IR.

Fiehn et al. [54] compared serum metabolomic profiles of obese patients and obese patients with T2DM. The researchers found that the levels of palmitate, heptadecane, stearate, oleate, and palmitoleic acid were significantly increased in obese patients with T2DM. Absolute or a relative lack of insulin in patients with T2DM leads to inefficient use of glucose and then the fatty acids become the main energy sources of β-oxidation. As a result, the levels of ketone bodies such as acetone, acetoacetate, and 3-hydroxybutyrate were significantly increased in T2DM patients [54]. Li X et al. [56] analyzed the plasma of patients with T2DM in a GC-GC and Time-of-Flight (TOF) method and five biomarkers with significant changes in contents were found. These biomarkers include 2-hydroxybutyric acid, linolenic acid, palmitic acid, stearic acid, and alpha-imidate. Liu et al. [67] performed metabolomic analysis of fatty acids in the serum of healthy subjects, T2DM patients, and postprandial hyperglycemia patients. The results showed that palmitic acid, stearic acid, oleic acid, linolenic acid, and linolenic acid were important biomarkers of T2DM. The concentrations of 3-hydroxybutyrate in the samples of plasma [54], serum [59,68], and urine [43] were higher in patients with T2DM than in normal subjects. In addition, Salek et al. [43] observed an increase of acetoacetate in patients.

Studies have found that T2DM may be associated with a reduced ability of skeletal muscles to oxidize free fatty acids in patients [69,70,71]. The mechanisms of mitochondrial oxidative fatty acid dysfunction and impaired insulin action are not fully understood. Adams et al. [72] used ultra-high performance liquid chromatography-mass spectrometry (UPLC-MS) technology to observe that acylcarnitine especially long-chain acylcarnitine (C10-carn, C12-carn, and C14-carn) increased significantly in T2DM subjects, which indicated the incomplete oxidation of long chain fatty acids. In addition, they demonstrated that C12-carn and C14-carn can significantly activate nuclear factor kappa B (NF-κB), induce inflammation, and play an important role in the development of IR [73,74]. For mitochondrial energy delivery, limited tricarboxylic acid (TCA) cycle activity leads to incomplete combustion of long-chain fatty acids and promotes accumulations of acyl carnitine by-products. The acylcarnitine by-products activate the NF-kB related pathway to inhibit insulin activity and the abnormal accumulation of acylcarnitine has been validated in targeted and untargeted metabolomic studies [75,76].

Phospholipids are key components of all cellular lipid bilayers and are involved in cellular signal transduction [77]. Jie Zhang et al. [78] applied automatic ultra-performance liquid chromatography-time-of-flight mass spectrometry (UPLC-TOF-MS) and multivariate statistical techniques for the study of T2DM. They found significant changes in the levels of leucine, dihydrosphingosine, and phytosphingosine in serum, which indicated disorders of amino acid metabolism and phospholipid metabolism in T2DM patients. Floegel et al. [48] used a targeted metabonomics approach in a large prospective study and many phospholipids (including sphingomyelin, 9 phosphatidyl cholines, and lysophosphatidylcholine C18:2) were found to be significantly associated with the occurrence of T2DM. Ha et al. [76] selected 53 male patients from a physical examination center who aged from 35 to 65 years old and had no history of cardiovascular disease, diabetes, or liver or kidney disease. In addition, 26 cases of newly diagnosed T2DM patients were treated as case groups. Another 27 non-diabetic subjects matched with BMI and age served as controls. They used UPLC-TOF-MS to process plasma samples. The subject’s characteristic curve was estimated and they found that decanoyl carnitine and lysolecithin (C14:0) were the best metabolites to predict the risk of T2DM.

Similarly, Wang-Sattler et al. [49] found that hemolytic phosphatidylcholine and acetylcarnitine can be biomarkers for prediabetes. Floegel et al. [48] used a directed metabonomics method in the study and found that the increase of diacylphosphatidylcholine as well as the reductions of alkylphosphatidylcholine and sphingomyelin were correlated with the occurrence of diabetes. Zhao et al. [79] used a non-targeted, high-resolution metabonomics study method for randomly selected 133 cases of new T2DM and 298 cased of non-T2DM in a family-based prospective study (SHFS). Seven biomarkers were found to predict the occurrence of T2DM. Among them, the phosphatidylcholine (PC22:6/20:4) belongs to the lipids group. Although the clear relationships between phospholipids and T2DM have not been elucidated, current studies suggest that the change in phospholipids may be an early factor in the pathogenesis of T2DM. Lee Y et al. [80] found that the cholesterol biosynthetic pathway was important in the development of T2DM. The T2DM group had a higher cholesterol level.

#### 4.1.3. Biomarkers Related to Carbohydrate Metabolism

Glucose is the main source of human energy. In the beginning of glycolysis, glucose is converted into pyruvate in cells. In aerobic conditions, pyruvate is converted to acetyl-CoA and then enters the TCA cycle to produce ATP. Lactate dehydrogenase catalyzes the conversion of pyruvate to lactic acid under hypoxic conditions. Lu J et al. [59] found that the serum pyruvate concentration in T2DM patients was higher than that in the normal control group, which indicates an increase in the glycolysis in T2DM patients. Messana et al. [81] found that the lactate levels in patients with T2DM were also significantly higher. The TCA cycle is also called the citric acid cycle. Messana et al. [81] demonstrated that there were higher levels of citric acid in patients with T2DM. The disorder of the TCA cycle is very complicated. In the study by Salek et al. [43], the increased blood citrate levels in T2DM patients were observed. However, in the urine of patients with T2DM, the levels of three TCA cycle intermediates, succinate, fumarate, and malate were significantly downregulated and further studies are needed to explain this phenomenon. 1,5-anhydroglucitol (1,5-AG) is a non-physiologically active polyol in the body. It has a structure similar to that of glucose. It is reabsorbed in the renal tubule, which is competitively inhibited by glucose. It maintains a steady state level through renal filtration and reabsorption at normal blood glucose levels [82,83]. However, with the increase of blood glucose concentration (>180 μmol/L), glucose cannot be completely reabsorbed by the kidney and it competitively inhibits the reabsorption of 1,5-AG by the renal tubules, which results in a decrease in serum 1,5-AG. Dungan et al. [84,85,86] found that 1,5-AG is a sensitive indicator of postprandial hyperglycemia [87,88,89] and many patients who are well-controlled by the glycemic control index HBc also have significant postprandial hyperglycemia [90].

In the above studies about metabolites in patients with T2DM, the trends of biomarkers detected by different researchers using different detection methods are not the same. We have summarized the results of some scholars [91,92,93]. According to statistical conclusions in reference, we found that *p* < 0.05 or *p* < 0.01 existed. As a result, there are statistically significant differences among the variations in metabolite content. The details are shown in Table 2.

### 4.2. Metabolic Pathway

**Figure 3:**
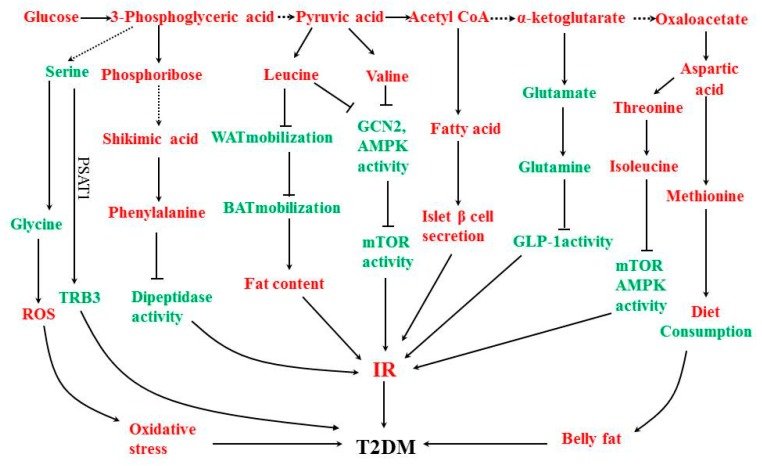
Metabolic pathway map.

Combining existing biochemical knowledge, the summarized biomarkers were analyzed and the following metabolic pathway maps were summarized, which is shown in Figure 3. Biomarkers are colored, the up-regulated ones are highlighted in red, and the down-regulated ones are shown in green. 

Combined with the analysis of existing biochemical knowledge and the results in Figure 3, the following six pathways were obtained.

#### 4.2.1. Serine Amino Acid Biosynthetic Pathway

When the blood glucose level increases, the amount of 3-phosphoglycerate converted from glucose through the glycolytic pathway increases. 3-phosphoglycerate produces serine after a series of reactions. Serine requires glutamic acid to create amino acids. However, reduced glutamate levels in T2DM patients results in the limited synthesis of serine. Decreased serine levels would reduce the production of glycine. As a synthetic precursor of glutathione, glycine is the most important endogenous antioxidant in the body. The decrease of its content will lead to an increase in ROS levels in the body, will result in an oxidative stress response, and will induce the formation of T2DM. At the same time, a decrease in serine content leads to a decrease in the expression of phospho-serine aminotransferase 1 (PSAT1) regulatory homolog 3 (TRB3) and results in IR [94]. Reduced glycine and serine levels would increase the risk of T2DM.

#### 4.2.2. Phosphate Pentose Pathway and Aromatic Amino Acid Biosynthesis Pathway

When the level of blood glucose increases, the phosphate ribose produced by the pentose phosphate pathway increases and the phosphate ribose produces shikimic acid through the shikimic acid pathway, which eventually increases the content of phenylalanine. Phenylalanine plays an important role as a precursor of phenylalanine derivatives. Phenylalanine derivatives act as dipeptide kinase inhibitors and can inhibit the activity of dipeptide kinase and protect incretin from degradation. Incretin promotes excess secretion of insulin from pancreatic islet β cells and increases the risk of IR and T2DM. The ribose phosphate can be further converted into histidine, which requires glutamine to provide an amide nitrogen into the histidine imidazole ring during histidine synthesis. Since the glutamine content decreases in T2DM patients, histidine synthesis is limited and the content is reduced. Histidine can alleviate oxidative stress and improve chronic inflammation. The increases of phenylalanine and histidine increase the risk of T2DM [95].

#### 4.2.3. Alanine Amino Acid Biosynthesis Pathway

Increased glucose levels lead to an increase in the pyruvate content through the glycolytic pathway. Pyruvate produces leucine and valine by a series of aminotransferases. An elevated leucine level leads to a reduction in fat mobilization mediated by a hormone-sensitive lipase (HSL) in white adipose tissue (WAT). The expression of uncoupling protein 1 (UCP1) in brown adipose tissue (BAT) was reduced and, when energy production decreased, the fat content increased. In addition to the association with regulating lipid homeostasis, the nutrient regulation signal induced by an increased leucine level down-regulates the mammalian rapamycin target protein (mTOR) pathway activity and reduces insulin sensitivity by inhibiting the amino acid receptor GCN2 and AMP-activated protein kinase (AMPK) activity, which results in T2DM. An increased valine level can also result in the reduction of fat mobilization and BAT heat production. It can inhibit mTOR and the AMPK signaling pathway, reduce insulin sensitivity, and increase the risk of T2DM [96].

#### 4.2.4. Fatty Acid Biosynthesis Pathway

Increased glucose levels lead to an increase in pyruvate content through the glycolytic pathway. Pyruvate produces acetyl-CoA through oxidative decarboxylation and acetyl-CoA increases the fatty acid content through a series of enzymatic reactions. Increased free fatty acids can cause defects in β-cell insulin secretion, increase the output of liver glycogen, reduce glucose uptake by muscle tissue, and, ultimately, increase the risk of developing T2DM.

#### 4.2.5. Glutamic Acid Amino Acid Biosynthetic Pathway

Increased glucose levels lead to increased α-ketoglutarate levels and α-ketoglutaric acid recombinates with free nitrogen (NH_4_^+^) to form glutamate under the action of glutamate dehydrogenase. Oxidative stress in patients with T2DM results in a large increase in RON content and a decrease in the content of NH_4_^+^ in cells. NH_4_^+^ is a raw material for the synthesis of glutamic acid. Its decrease in the synthesis of glutamate results in a restricted synthesis of glutamate. The reduction of glutamate content reduces glutamine production by glutamine synthetase. Glutamine can increase the secretion of glucagon-like peptide-1 (GLP-1) and increase the level of insulin in blood. Decreased glutamine levels lead to insufficient insulin secretion and also lead to T2DM.

#### 4.2.6. Aspartate Amino Acid Biosynthetic Pathway

Oxaloacetic acid produces aspartic acid under the action of transaminase. Aspartic acid can produce methionine and isoleucine after a series of complicated reactions. Methionine may increase dietary intake and reduce energy consumption, increase belly fat, and reduce insulin sensitivity [97]. Similar to leucine, an increase in the isoleucine level results in a decrease in fat mobilization and a decrease in BAT heat production by inhibiting mTOR and the AMPK signaling pathway and reducing insulin sensitivity [96]. Increased levels of tyrosine and isoleucine increase the risk of T2DM.

## 5. Discussion

Recently, with the application and rapid development of metabonomics in the study of diabetes, the knowledge of the pathogenesis of diabetes has turned into totalization and systematization. This provides scholars with new ideas and methods to study the prevention and treatment methods of diabetes. Metabonomics seeks biomarkers that can reflect the pathophysiological processes of T2DM by detecting changes in a large number of endogenous metabolites in the body. It also creates new opportunities for diabetes prevention and treatment.

Current research shows that the metabolites of amino acids, lipids, and carbohydrates are closely related with the occurrence and development of diabetes. Amino acids associated with diabetes mainly include three kinds of branched chain amino acids and two kinds of aromatic amino acids and their metabolites. In addition, 2-AAA, 2-HB, α-HB, glycine and its metabolites, and enzymes related to glycine are also important substances that cause T2DM. Lipids that influence diabetes mainly include phosphoglycerides (such as phosphatidylcholine, phosphatidylethanolamine), and lipid derivatives (such as phytosphingosine, dihydrosphingosine). When it comes to sugar, it mainly includes intermediate products of the TCA cycle such as citric acid and malic acid.

Metabolic pathways enriched according to the aforementioned biomarkers also point to the pathogenesis of T2DM, which contains the phosphate pentose pathway, the serine amino acid biosynthetic pathway, the aromatic amino acid biosynthesis pathway, the alanine amino acid biosynthesis pathway, the fatty acid biosynthesis pathway, the glutamic acid amino acid biosynthetic pathway, and the aspartate amino acid biosynthetic pathway. In addition, abnormal metabolism of glucose through a series of metabolic pathways eventually leads to a greater likelihood of T2DM occurring.

Scholars use metabonomics technology to search for specific biomarkers of diabetes and enriched metabolic pathways based on biomarkers. This will not only provide clinical experimental evidence for the early diagnosis and active prevention and treatment of diabetes, but will also provide ideas and references for developing anti-diabetic medicine. In general, the application and popularization of metabonomics technology in diabetes research will bring about a significant revolutionary change in basic research as well as clinical prevention and treatment of diabetes.

## Figures and Tables

**Table 1:** Summary of the advantages and disadvantages of NMR and MS.

Characteristic	NMR	MS
Objectivity	yes	yes
Repeatability	fine	fine
Sensibility	general	selective sensitivity
Resolution	general	general
Flux	high	high
Sample preparation	little	a large number
Molecular information	abundant	general
Labor intensity	low	general
Recurrent costs	low	high
Single sample cost	low	general

**Table 2:** List of altered metabolic pathways in T2DM patients.

Pathway	Metabolite	Change of Direction (vs. Healthy Control)	Sample	Platform	Reference
Amino acid	Leucine	Up	Serum	NMR,UPLC-MS,GC-MS	[68]
metabolism		Up	Plasma	UPLC-MS	[76]
		Up	Plasma	GC-MS	[54]
	Isoleucine	Up	Serum	NMR,UPLC-MS,GC-MS	[68]
	Valine	Up	Serum	NMR,UPLC-MS,GC-MS	[68]
		Up	Serum	GC-MS	[45]
	Glutamine	Up	Urine	NMR	[43]
		Up	Serum	GC-MS	[59]
	Glutamique	Down	Serum	NMR	[44]
	lysine	Down	Plasma	GC-MS	[54]
		Down	Serum	NMR	[44]
		Down	Serum	GC-MS	[45]
	Glysine	Down	Plasma	GC-MS	[54]
		Down	Serum	LC-MS	[49]
		Down	Serum	LC-MS	[48]
		Down	Plasma	LC-MS/MS	[47]
	2-Hydroxybutyrate	Up	Plasma	GC*GC-MS	[56]
		Up	Plasma	GC-MS	[54]
		Up	Serum	GC-MS	[59]
		Up	Plasma	GC-MS	[54]
	Serine	Down	Plasma	UPLC-MS	[76]
	Tyrosine	Down	Serum	NMR	[44]
	Phenylalanine	Up	Plasma	UPLC-MS	[76]
		Up	Plasma	LC-MS/MS	[47]
		Up	Serum	GC-MS	[59]
		Up	Serum	LC-MS	[48]
	Tryptophan	Down	Urine	NMR	[43]
	Alanine	Down	Serum	NMR	[44]
	Methionine	Up	Serum	GC-MS	[59]
	Histidine	Down	Serum	NMR	[44]
		Down	Urine	NMR	[43]
	Hippurate	Up	Urine	NMR	[81]
	Taurine	Up	Urine	NMR	[43]
		Up	Plasma	LC-MS/MS	[47]
Lipid	3-Hydroxybutyrate	Up	Plasma	GC-MS	[54]
metabolism		Up	Serum	NMR,UPLC-MS,GC-MS	[68]
		Up	Urine	NMR	[43]
		Up	Serum	GC-MS	[59]
	Acetoacetate	Up	Urine	NMR	[43]
	Fatty acids	Up	Plasma	GC-MS	[54]
		Up	Serum	GC-MS	[59]
	Lyso PCs	Up	Plasma	UPLC-MS	[76]
		Up	Plasma	UPLC-MS	[76]
	Lyso PC (18:2)	Down	Serum	LC-MS	[49]
		Down	Serum	LC-MS	[48]
	Lyso PEs	Up/Down	Plasma	UPLC-MS	[76]
		Up/Down	Plasma	UPLC-MS	[76]
	PCs Acetylcarnitines	Up/Down	Serum	LC-MS	[48]
		Up	Plasma	UPLC-MS	[76]
		Up	Plasma	UPLC-MS	[75]
		Up	Plasma	UPLC-MS	[72]
		Up	Plasma	UPLC-MS	[76]
	Palmitic acid	Up	Plasma	GC-MS	[66]
		Up	Plasma	GCxGC-TOFMS	[56]
		Up	Serum	GC-MS	[67]
	linolenic acid	Up	Plasma	GCxGC-TOFMS	[56]
	Dihydrosphingosine	Down	Serum	UPLC-oaTOF	[78]
	Phytosphingosine	Down	Serum	UPLC-oaTOF	[78]
	cholesterol	Up	Serum	LC-MS	[80]
Carbohydrate	citric acid	Up	Urine	NMR	[81]
metabolism		Up	Urine	NMR	[43]
	1,5-Anhydrogluticol	Down	Serum	NMR,UPLC-MS,GC-MS	[68]
		Down	Serum	GC-MS	[59]
	Pyruvate	Down	Serum	GC-MS	[59]
	Lactate	Up	Serum	GC-MS	[59]
	Malate	Down	Urine	NMR	[43]
	Succinate	Down	Urine	NMR	[43]
	Fumarate	Down	Urine	NMR	[43]
